# Investigating the duality of *Inpp4b* function in the cellular transformation of mouse fibroblasts

**DOI:** 10.18632/oncotarget.27293

**Published:** 2019-10-29

**Authors:** Emily Marie Mangialardi, Keyue Chen, Brittany Salmon, Jean Vacher, Leonardo Salmena

**Affiliations:** ^1^Department of Pharmacology and Toxicology, University of Toronto, Toronto, Ontario, Canada; ^2^Institut de Recherches Cliniques de Montréal, Département de Médecine, Université de Montréal, Montréal, Québec, Canada; ^3^Princess Margaret Cancer Centre, University Health Network, Toronto, Ontario, Canada

**Keywords:** Inpp4b, MEF, RAS, E1A, SV40-T-Large

## Abstract

*Inositol Polyphosphate 4-Phosphatase, Type II (INPP4B)* is a tumour suppressor in breast, ovarian, prostate, thyroid and other cancers, attributed to its ability to reduce oncogenic Akt-signaling. However, emerging studies show that *INPP4B* also has tumour-promoting properties in cancers including acute myeloid leukemia, colon cancer, melanoma and breast cancer. Together these findings suggest that *INPP4B* may be a context dependent cancer gene. Whether *INPP4B* functions solely in a tumour suppressing or tumour promoting manner, or both in non-transformed cells is currently not clear. In this study, consequences of deficiency and overexpression of *INPP4B* on cellular transformation was investigated using a mouse embryonic fibroblast (MEF) model of cellular transformation. We observed that neither deficiency nor overexpression of *INPP4B* was sufficient to induce neoplastic transformation, alone or in combination with *H-Ras*^*V12*^ or *E1A* overexpression. However, *Inpp4b*-deficiency did cooperate with *SV40 T-Large*-mediated cellular transformation, a finding which was associated with increased phosphorylated-Akt levels. Transformation and phosphorylated-Akt levels were dampened upon overexpression of *INPP4B* in *SV40 T-Large*-MEF. Together, our findings support a model where INPP4B function suppresses transformation mediated by *SV40* *T-Large*, but is inconsequential for *Ras* and *E1A* mediated transformation.

## INTRODUCTION

The phosphoinositide-3-kinase (PI3K) signaling pathway regulates cell survival, proliferation, metabolism and various other processes linked to cell growth. Human cancers frequently acquire mutations resulting in aberrant activation of PI3K signaling which is often associated with increased tumour progression and resistance to cancer therapies. The PI3K pathway is activated by growth-factor mediated activation of receptor tyrosine kinases (RTKs) and G protein-coupled receptors (GPCRs) which transduce signals through class I PI3K via phosphorylation of the 3-hydroxyl group of the inositol ring of phosphatidylinositol-4,5-bisphosphate [PtdIns(4,5)P_2_] to generate phosphatidylinositol-3,4,5-trisphosphate (PtdIns(3,4,5)P_3_ [PIP_3_]) [1, 2]. PIP_3_ is subsequently dephosphorylated by either PTEN to form phosphatidylinositol-4,5-bisphosphate [PtdIns(4,5)P_2_] or by SHIP phosphatases to form phosphatidylinositol-3,4-bisphosphate [PtdIns(3,4)P_2_]. The 3’phosphorylated phosphatidylinositols like PIP_3_ and PtdIns(3,4)P_2_ recruit cytosolic proteins containing pleckstrin homology (PH) domains, such as the serine/threonine kinase Akt. Once recruited to membranes, the kinase activity of Akt is activated by phosphorylation to promote downstream signaling to cell survival, growth, proliferation, cell migration and angiogenesis, through phosphorylation of specific intracellular protein substrates [3].

*Inositol Polyphosphate 4-Phosphatase, Type II (INPP4B)*, a lipid phosphatase that preferentially hydrolyzes PtdIns(3,4)P_2_ to generate phosphatidylinositol-3-monophosphate [PtdIns(3)P], was characterized as a gene with a key role in PI3K pathway signaling [4]. Since both PtdIns(3,4)P_2_ and PIP_3_ promote Akt recruitment to the plasma membrane, INPP4B was predicted to act as a tumour suppressor by inhibiting Akt recruitment, activation and thus downstream PI3K pathway signaling [5–8]. The first evidence characterizing *INPP4B* as a gene with importance in cancer was from a RNA interference screen in immortalized human mammary epithelial cells (HMEC) designed to identify candidate tumour suppressors [9]. Several studies have subsequently validated a tumour suppressor role for *INPP4B* in breast, ovarian, skin, and prostate cancer among others [10, 4, 8, 11–23]. In these studies, *INPP4B* loss resulted in elevated Akt activation, increased cell survival and a more aggressive growth phenotypes associated with poor outcomes for cancer patients [13, 16, 24]. These findings for INPP4B contribute to the increasing role of phosphoinositide phosphatases other than PTEN in cancer; these include the INPP5-family members such as INPP5J/PIPP, INPP5D/SHIP1, INPPL1/SHIP2, and INPP5E [25–29]. Notably, despite the abundance of clinical data supporting a tumour suppressor role for INPP4B, there is no evidence that *Inpp4b* deletion alone in mouse models leads to tumour formation [17, 19, 30]. However when *Inpp4b* loss was combined with *Pten* heterozygosity, it altered the penetrance of the Pten-spectrum of tumours, and notably malignant thyroid cancer was observed [17, 19, 30]. Thus it has been suggested that INPP4B may be a tumour suppressor in the context of PTEN loss, and may have weak tumour suppressive function otherwise [31].

Conversely, emerging findings in malignancies including acute myeloid leukemia (AML), colon cancer, melanoma and breast cancer among others suggest that overexpression of *INPP4B* is also associated with promoting aggressive cancer phenotypes [32–36]. Signaling downstream of PtdIns(3)P has been explored as a possible mechanism. For instance, PtdIns(3)P mediated activation of Serum/Glucocorticoid Regulated Kinase Family Member 3 (SGK-3) was observed downstream of INPP4B overexpression in some cancers [34, 36–39]. Moreover, PtdIns(3)P has very important cellular roles, which include endosomal trafficking and autophagy which are currently unexplored in the context of INPP4B overexpression [40]. Moreover, *INPP4B* was reported to have both tumour promoting and tumour suppressing features in different subsets of the same cancer. For instance in melanoma and breast cancer, both *INPP4B* loss and *INPP4B* overexpression were associated with downstream oncogenic signaling through Akt and SGK3, respectively [8, 37, 38, 41]. Altogether, these findings point to a putative contextual role for *INPP4B* in cancer [42, 43]. Nevertheless, mechanisms underlying the context-dependent cancer functions of INPP4B remain to be elucidated.

A growing body of evidence links altered levels of *INPP4B* expression to the progression of cancer. However, a role for INPP4B in the transformation of primary cells remains unexplored. Herein, we sought to investigate the consequences of *Inpp4b*-deficiency and *INPP4B*-overexpression on the cellular transformation of primary MEF in combination with oncogenic drivers including *H-Ras*^*V12*^, *E1A* or *SV40 T-Large*. Exploring a role for *Inpp4b* in MEF transformation may provide insight on whether co-operating driver mutational signaling will alter *Inpp4b* context dependent outcome in tumourigenesis.

## RESULTS

### Characterization of primary *Inpp4b*^*+/+*^, *Inpp4b*^*+/-*^ and *Inpp4b*^*-/-*^ MEF

**Figure 1:**
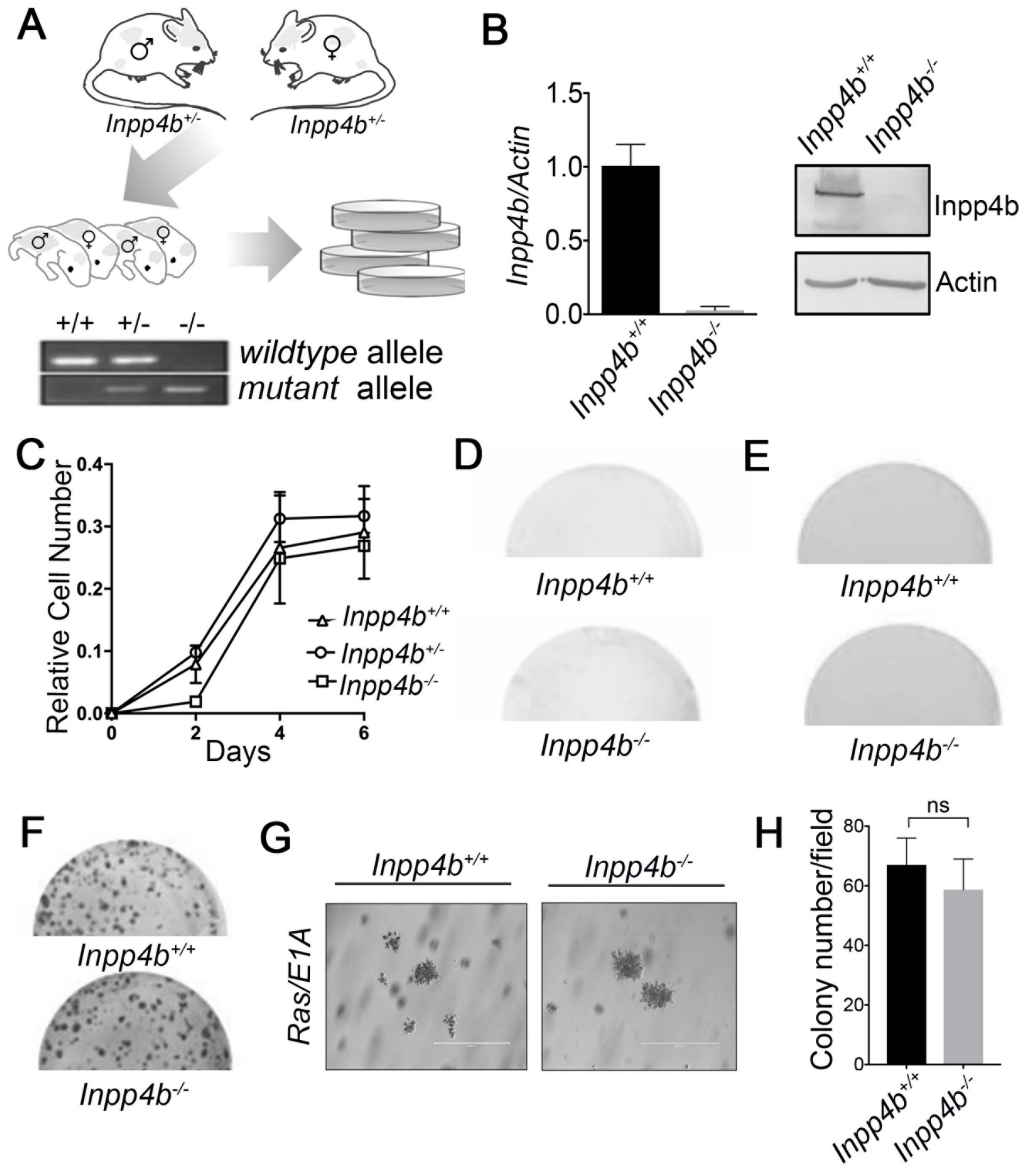
Generation and characterization of primary *Inpp4b*^*+/+*^, *Inpp4b*^*+/-*^ and *Inpp4b*^*-/-*^ MEF. (**A**) Schematic illustrating breeding strategy for generation of primary MEF and a typical *Inpp4b* genotyping PCR result is illustrated. (**B**) RT-qPCR and Immunoblot demonstrating Inpp4b expression levels in *Inpp4b*^*+/+*^ and *Inpp4b*^-/-^ MEF. (**C**) Growth curve, (**D**) colony formation (**E**) and soft agar assay of primary *Inpp4b*^*+/+*^ and *Inpp4b*^-/-^ MEF. (**F**) Colony formation assay with *Inpp4b*^*+/+*^ and *Inpp4b*^-/-^ MEF empty vector and *12SLRC H-Ras*^*V12*^ */E1A* infection. (**G**) Representative soft agar assay of *12SLRC H-Ras*^*V12*^ */E1A* infected *Inpp4b*^*+/+*^ and *Inpp4b*^-/-^ MEF. Photos of representative colonies at 20X. (**H**) Quantitation of quantitation of foci formation assay.

To investigate the role of *Inpp4b* loss on cellular transformation we generated E13.5 MEF from a constitutive *Inpp4b* exon 10 knockout (*Inpp4b*^*-/-*^) mouse model [30]. PCR genotyping of cultured MEFs derived from timed-matings of *Inpp4b*^*+/-*^ mice was performed to determine *Inpp4b*^*+/+*^ *, Inpp4b*^*+/-*^ *or Inpp4b*^*-/-*^ genotypes (Figure 1A). RT-QPCR and immunoblots were performed to validate loss of both transcript and protein levels of Inpp4b in *Inpp4b*^*-/-*^ MEF (Figure 1B). Growth characteristics of primary MEF was evaluated in short term growth assays where we observed no significant differences in the mean growth rates of *Inpp4b*^*+/+*^ *, Inpp4b*^*+/-*^ *and Inpp4b*^*-/-*^ MEF (Figure 1C). Similarly, long term clonogenic growth potential was tested in primary *Inpp4b*^*+/+*^ *and Inpp4b*^*-/-*^ MEF. After 11 days of growth, only sparse spontaneous clone formation was observed in both *Inpp4b*^*+/+*^ and *Inpp4b*^*-/-*^ MEF, with no measurable difference between genotypes (Figure 1D). Finally, neither *Inpp4b*^*+/+*^ nor *Inpp4b*^*-/-*^ MEF were observed to grow as anchorage independent colonies in soft agar (Figure 1E).

To investigate the necessity for *Inpp4b* in cellular transformation, we examined the consequences of *Inpp4b* deficiency on *H-Ras*^*V12*^ */E1A* mediated MEF transformation. For these experiments we infected early passage *Inpp4b*^*+/+*^ and *Inpp4b*^-/-^ MEF with retrovirus expressing both *H-Ras*^*V12*^ and *E1A* (*12SLRC*). Upon infection of cells, we observed no difference in the morphology in all *H-Ras*^*V12*^ */E1A* transformed cells of either genotype. Moreover, we observed no difference between the transformed MEF from *Inpp4b*^*+/+*^ and *Inpp4b*^-/-^ backgrounds on foci formation in clonogenic assays (Figure 1F) and anchorage independent colonies in soft agar (Figure 1G, 1H). These data suggest that *Inpp4b-*deficiency does not lead to spontaneous transformation of MEF and that *Inpp4b* expression is dispensable for *H-Ras*^*V12*^ */E1A* mediated MEF transformation.

### Neither loss nor overexpression of *INPP4B* cooperate with *H-Ras*^*V12*^ in *MEF* transformation

**Figure 2:**
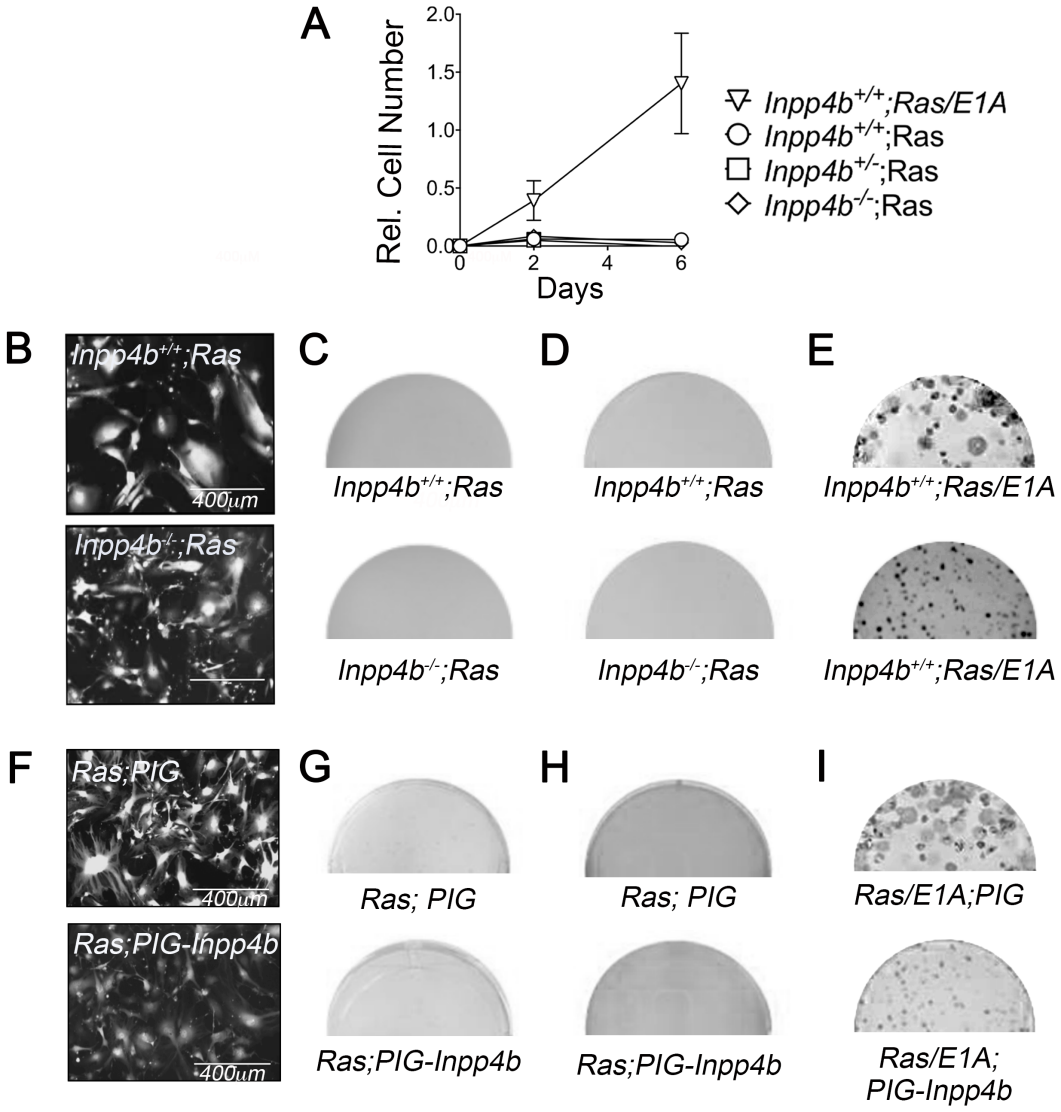
*Inpp4b* loss or overexpression do not cooperate with *H-Ras*^*V12*^ in MEF transformation. (**A**) 6-day growth curve of *Inpp4b*^*+/+*^, *Inpp4b*^+/-^ and *Inpp4b*^-/-^ MEF after *HRas*^*V12*^ infection. *12SLRC H-Ras*^*V12*^ */E1A* infected *Inpp4b*^*+/+*^ MEF used as control. (**B**) Morphology of eGFP-expressing *Inpp4b*^*+/+*^ and *Inpp4b*^-/-^ MEF after *H-Ras*^*V12*^ infection. (**C**) Colony formation and (**D**) Soft agar assay of primary *Inpp4b*^*+/+*^ and *Inpp4b*^-/-^ MEF after *H-Ras*^*V12*^ infection. (**E**) *12SLRC H-Ras*^*V12*^ */E1A* infected colony assay (top) and soft agar (bottom) controls. Full wells are depicted. (**F**) Morphology of eGFP-expressing *Inpp4b*^*+/+*^ MEF after *H-Ras*^*V12*^ and *PIG* or *PIG-Inpp4b* infection. (**G**) Colony formation and; (**H**) Soft agar assay of primary *Inpp4b*^*+/+*^ MEF after *H-Ras*^*V12*^ and *PIG* or *PIG-Inpp4b* infection (**I**) *12SLRC H-Ras*^*V12*^ */E1A* and *PIG* or *PIG-Inpp4b* infected colony assay (top) and soft agar (bottom) controls.

To characterize the cooperativity of *Inpp4b*-deficiency with *H-Ras*^*V12*^ overexpression in cellular transformation, early passage *Inpp4b*^*+/+*^ and *Inpp4b*^-/-^ MEF were infected with retroviral particles generated from the *pBabe-H-Ras*^*V12*^ *-puro* vector. Morphologically, both *H-Ras*^*V12*^ *; Inpp4b*^*+/+*^ *H-Ras*^*V12*^ *; Inpp4b*^-/-^ MEF demonstrated the expected features of oncogene-induced senescence (OIS) including complete growth inhibition (Figure 2A) and large multinucleated senescent-like cells (Figure 2B). Neither *H-Ras*^*V12*^ *; Inpp4b*^*+/+*^ nor *H-Ras*^*V12*^ *; Inpp4b*^-/-^ MEF were able to form colonies in clonogenic assays (Figure 2C). Similarly, no anchorage-independent colonies were observed in soft agar (Figure 2D). Conversely, positive control MEF infected with *H-Ras*^*V12*^ and *E1A* retroviruses generated rapidly proliferating cells (Figure 2A), abundant foci (Figure 2E, top) and numerous anchorage independent colonies (Figure 2E, bottom).

Next, to examine the potential of *INPP4B* as an oncogene in MEF, we investigated whether *INPP4B*-overexpression could cooperate with *H-Ras*^*V12*^ in cellular transformation. Retrovirus generated from *pWZL-H-Ras*^*V12*^ *-hygro* vector was combined with either *MSCV-Puro-IRES-GFP (PIG)-empty* or *PIG-INPP4B* retrovirus to co-infect *wild-type* MEF followed by selection with hygromycin B and puromycin. Morphologically both *H-Ras*^*V12*^ *; PIG* or *H-Ras*^*V12*^ *; PIG-INPP4B* infected MEF appeared large and multinucleated (Figure 2F) as expected with *H-Ras*^*V12*^ infection. Foci were not observed in clonogenic assays (Figure 2G) and similarly, no anchorage-independent colonies were observed in soft agar assays (Figure 2H). *Inpp4b*^*+/+*^ MEF infected with *H-Ras*^*V12*^ and *E1A* retroviruses which generated abundant foci (Figure 2I, top) and anchorage independent colonies (Figure 2I, bottom) were used as positive controls. Overall, neither deficiency nor overexpression of *INPP4B* demonstrated cooperativity with *H-Ras*^*V12*^-overexpression in cellular transformation.

### Neither loss nor overexpression of *INPP4B* cooperate with *E1A* in *MEF* transformation

**Figure 3:**
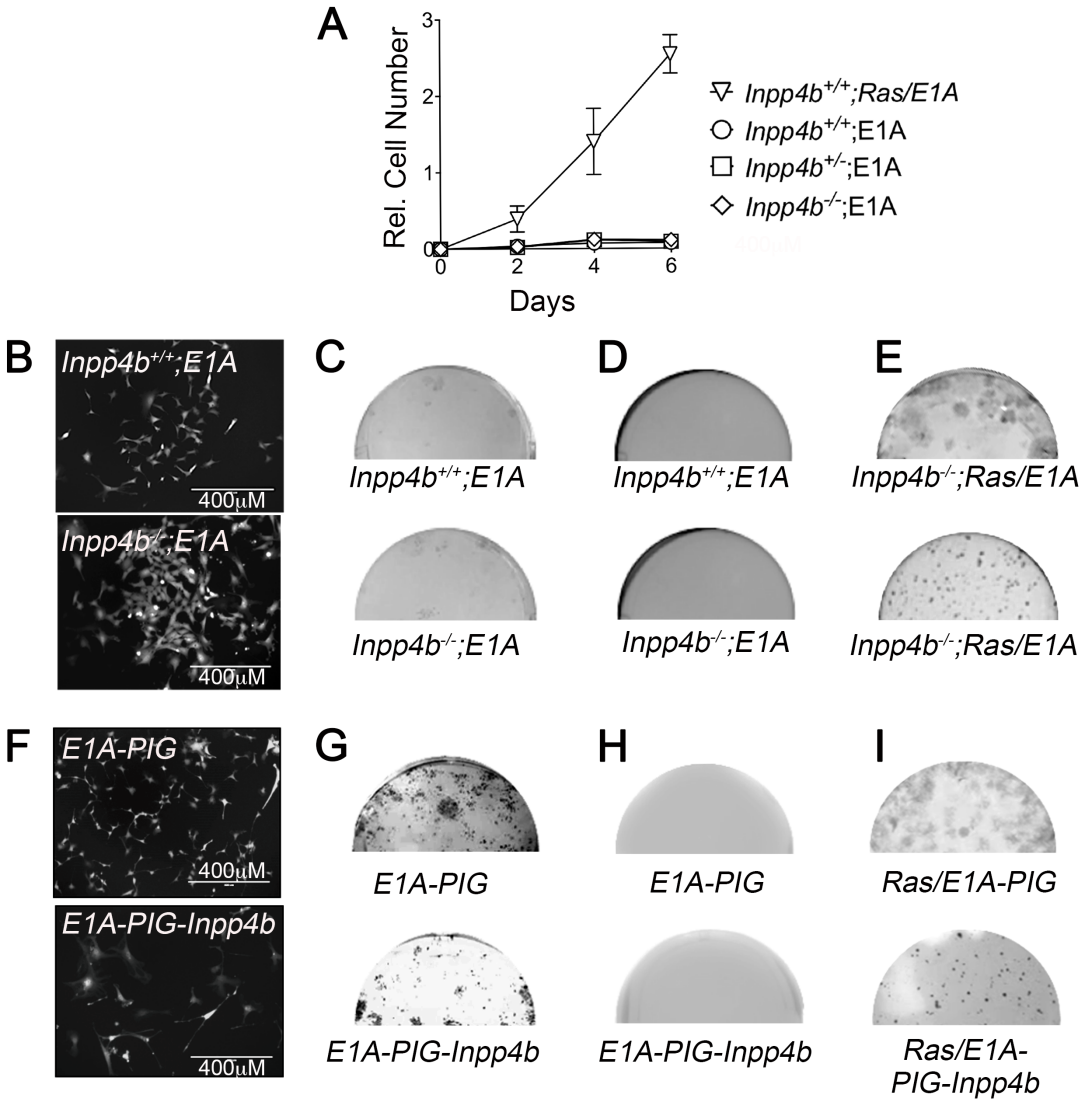
*Inpp4b* loss or overexpression do not cooperate with *E1A* in MEF. (**A**) 6-day growth curve of MEF after *E1A* infection. *12SLRC H-Ras*^*V12*^ */E1A* infected *Inpp4b*^*+/+*^ MEF used as control. (**B**) Morphology of eGFP-expressing *Inpp4b*^*+/+*^ and *Inpp4b*^-/-^ MEF after *E1A* infection. (**C**) Colony formation and; (**D**) Soft agar assay of primary *Inpp4b*^*+/+*^ and *Inpp4b*^-/-^ MEF after *E1A* infection and (**E**) *12SLRC H-Ras*^*V12*^ */E1A* infected colony assay (left) and soft agar (right) controls. Half wells are depicted. (**F**) Morphology of eGFP-expressing *Inpp4b*^*+/+*^ MEF after *E1A* and *PIG* or *PIG-Inpp4b* infection. (**G**) Colony formation and; (**H**) Soft agar assay of primary *Inpp4b*^*+/+*^ MEF after *E1A* and *PIG* or *PIG-Inpp4b* infection (**I**) *12SLRC H-Ras*^*V12*^ */E1A* and *PIG* or *PIG-Inpp4b* infected colony assay (left) and soft agar (right) controls.

To investigate whether loss of *Inpp4b* is sufficient to cooperate with *E1A* in promoting cellular transformation, early passage *Inpp4b*^*+/+*^ and *Inpp4b*^-/-^ MEF were infected with *pWZL-E1A-hygro* retrovirus and selected with hygromycin B. Immediately after selection, MEF were plated for growth analysis, clonogenic assay and soft agar assays. *Inpp4b*^*+/+*^, *Inpp4b*^+/-^ and *Inpp4b*^-/-^ MEF infected with *E1A* alone were growth inhibited compared to *H-Ras*^*V12*^; *E1A* controls (Figure 3A). Morphologically both *Inpp4b*^*+/+*^ and *Inpp4b*^-/-^ MEF appeared smaller and more dispersed compared to primary MEF (Figure 3B); clonal outgrowth on plastic observed after 21 days was similarly very minimal in both *Inpp4b*^*+/+*^ and *Inpp4b*^-/-^ MEF (Figure 3C) and no anchorage independent growth colonies formed in soft agar (Figure 3D). Conversely, *Inpp4b*^*+/+*^ and *Inpp4b*^-/-^ MEF overexpressing both *H-Ras*^*V12*^ and *E1A* rapidly proliferated in 6-day growth curves (Figure 3A), grew many more foci in the clonogenic assay (Figure 3E, top) and formed large robust colonies in soft agar (Figure 3E, Bottom).

Next, *INPP4B* overexpression was tested for its ability to cooperate with *E1A* to induce cellular transformation in MEF. For these experiments, *wild-type* MEF were retrovirally infected with *pWZL-E1A-hygro* vector with either *PIG* or *PIG-INPP4B* and selected with hygromycin B and puromycin for 48 hours. Morphologically, *E1A; PIG* MEF appeared smaller than primary cells (Figure 3F, top) whereas *E1A; PIG-INPP4B* MEF appeared larger, more elongated with long spindle-like protrusions and multinucleated (Figure 3F, bottom). No significant differences in foci counts were observed between *E1A; PIG* and *E1A; PIG-INPP4B* in clonogenic assays (Figure 3G). Anchorage independent growth was not observed in either *E1A; PIG* and *E1A; PIG-INPP4B* MEF (Figure 3H), but both clones and foci were clearly observed in the control cells infected with *H-Ras*^*V12*^ and *E1A* retroviruses (Figure 3I). Overall, as with *H-Ras*^*V12*^-overexpression, neither *Inpp4b-*deficiency nor *INPP4B*-overexpression were able to cooperate with *E1A* in cellular transformation.

### *Inpp4b* deficiency increases colony forming potential in *SV40 T-Large MEF*

**Figure 4:**
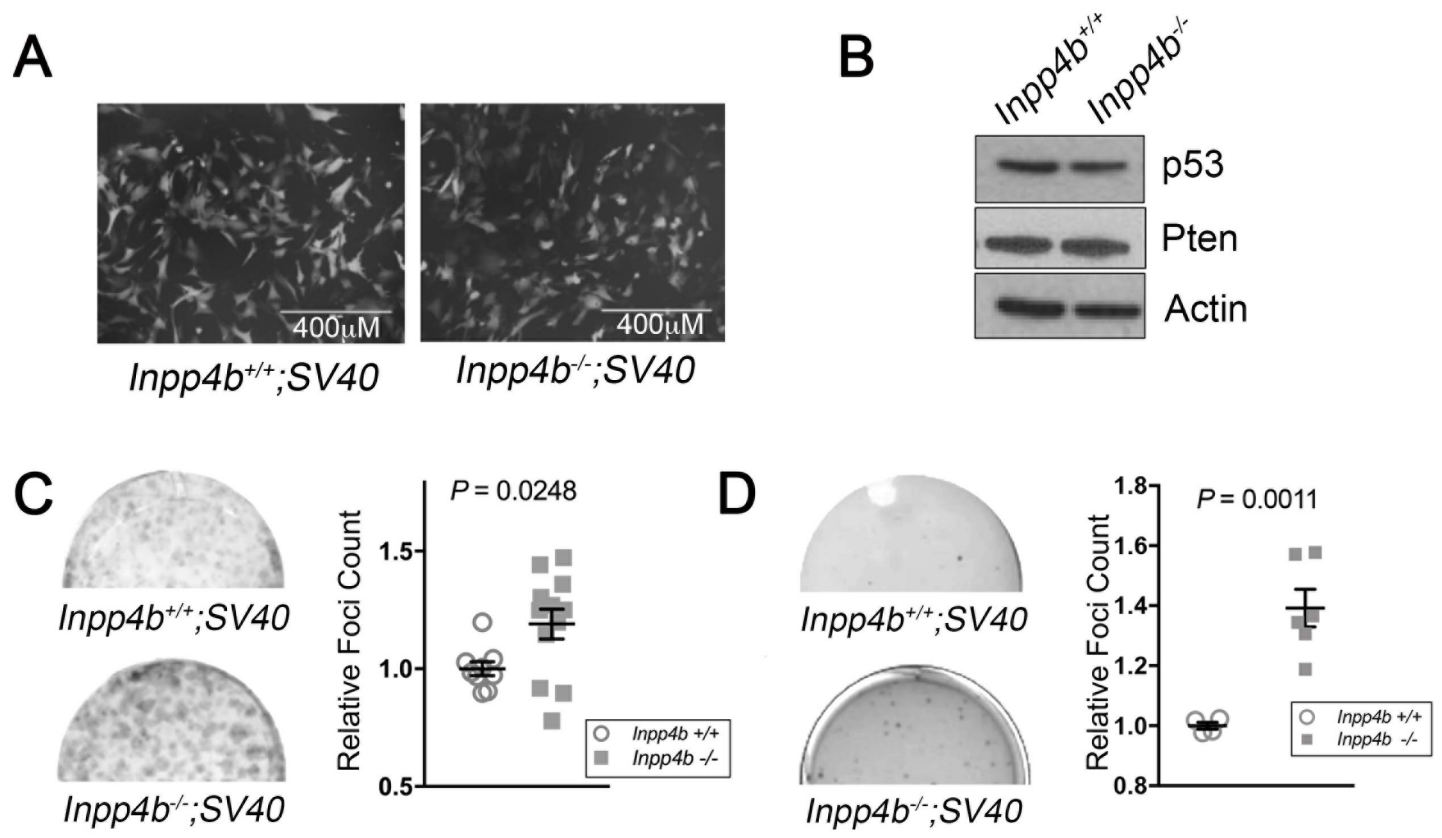
*Inpp4b* deficiency increases colony forming potential of *SV40 T-Large* in MEF. (**A**) Morphology of eGFP-expressing *Inpp4b*^*+/+*^ and *Inpp4b*^-/-^ MEF after *SV40 T-Large* infection. (**B**) Immunoblot of *Inpp4b*^*+/+*^ and *Inpp4b*^-/-^ *SV40 T-Large* MEF. (**C**) Representative clonogenic and (**D**) Soft agar assay plates with quantitation below. *P*-values were determined using the Student’s *t*-test.

We next investigated cooperativity of *Inpp4b*-deficiency with *SV40 T-large* overexpression. As before, *SV40 T-Large* retrovirus was used to infect early passage *Inpp4b*^*+/+*^ and *Inpp4b*^-/-^ MEF. Morphologically, no differences were observed between *Inpp4b*^*+/+*^ and *Inpp4b*^-/-^ MEF lines (Figure 4A). Immunoblot analysis of *SV40 T-Large* *Inpp4b*^*+/+*^ and *Inpp4b*^-/-^ demonstrated similar levels of p53 protein and no changes in PTEN expression (Figure 4C). Notably, unlike *H-Ras*^*V12*^ and *E1A*, *SV40 T-Large* infected *Inpp4b*^-/-^ MEF demonstrated a significant (*P* = 0.025) increase in foci formation (Figure 4C) and a significant (*P* = 0.0011) increase in anchorage independent colonies in soft agar (Figure 4D). These findings demonstrate cooperativity between *SV40 T-Large* overexpression *and Inpp4b* deficiency in MEF and suggest that *Inpp4b* may function as a tumour suppressor in the context of *SV40 T-Large* transduction.

**Figure 5:**
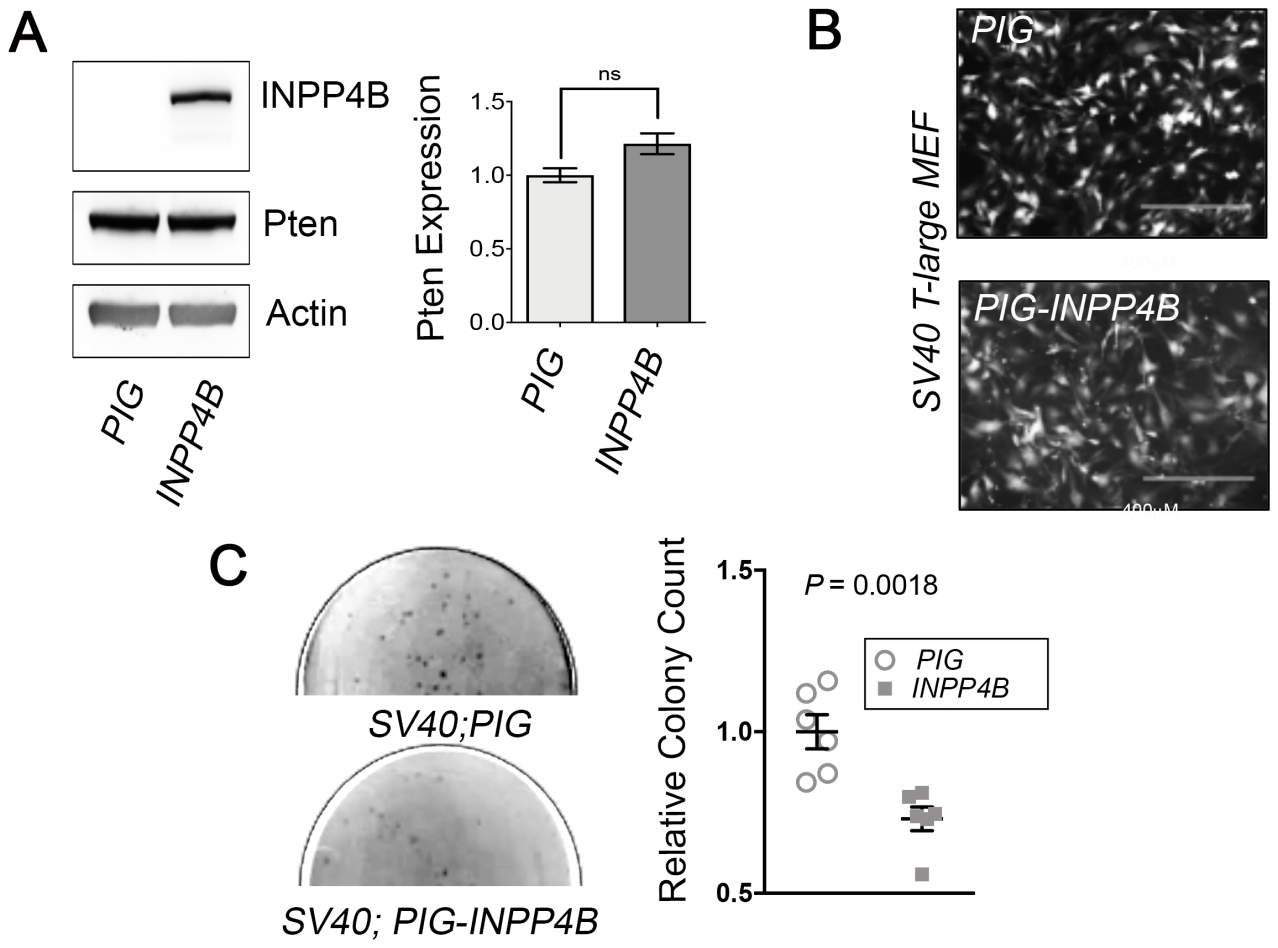
*Inpp4b* overexpression decreases transformation potential in *SV40 T-Large* MEF. (**A**) Immunoblot measuring INPP4B overexpression and Pten levels in *SV40T-Large* MEF (*n* = 5). (**B**) Morphology of eGFP-expressing *Inpp4b*^*+/+*^ *SV40T-Large* MEF with *PIG* or *PIG-Inpp4b* transduction. (**C**) Representative soft agar assay plates and quantitation of *Inpp4b*^*+/+*^ *SV40T-Large* MEF with *PIG* or *PIG-Inpp4b* colonies. *P*-values were determined using the Student’s *t*-test.

Given this increase in *SV40 T-Large* transformation potential observed in *Inpp4b*^-/-^ MEF, we reasoned that *INPP4B* overexpression would limit anchorage independent growth of *SV40 T-Large* MEF. To investigate this, early passage *SV40 T-Large MEF* were transduced with *PIG* and *PIG-INPP4B* retrovirus (Figure 5A). Although there were no gross morphological differences between *PIG* and *PIG-INPP4B* transduced *SV40 T-Large* MEF (Figure 5B), we did observe that *INPP4B* overexpression in MEF SV*40 T-Large* MEF displayed a significant reduction (*P* = 0.0018) in colony number as compared to control (Figure 5C). Immunoblot analysis of *SV40 T-Large* *Inpp4b*^*+/+*^ and *Inpp4b*^-/-^ demonstrated no changes Pten protein levels upon INPP4B overexpression (Figure 5A).

### *Inpp4b* modulates EGF stimulated PI3K signaling in *SV40 T-Large* MEF

**Figure 6:**
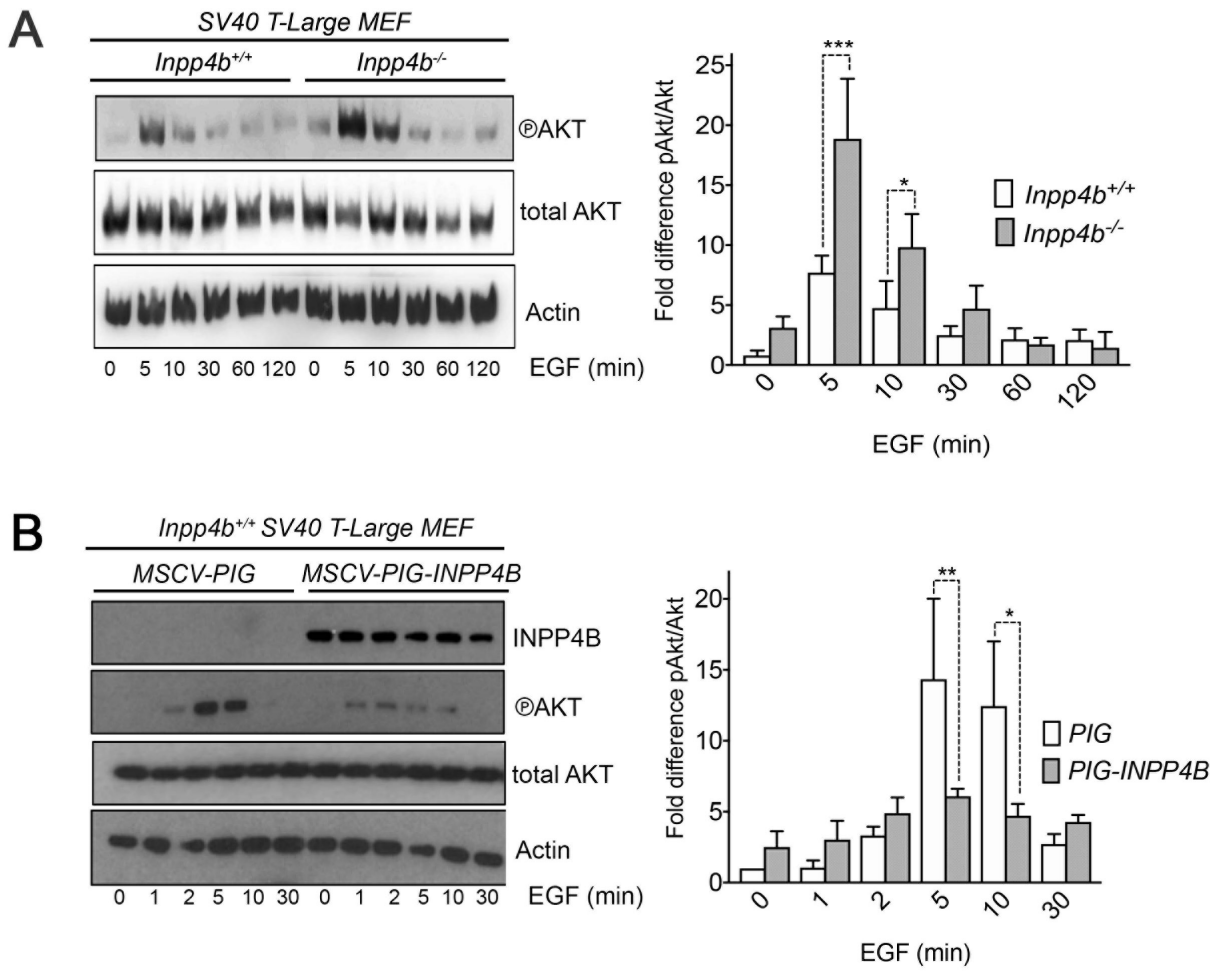
*Inpp4b* modulates EGF stimulated Akt activation in *SV40 T-Large* MEF. SV40T-Large MEF were serum starved and stimulated with 100 ng/mL of EGF and immunoblotted with pSer^473^-Akt. (**A**) Immunoblot of pSer^473^-Akt of *SV40 T-Large* *Inpp4b*^*+/+*^ and *Inpp4b*^-/-^ MEF with quantitation of pSer^473^-Akt activation status (*n* = 4) (**B**) Immunoblot of pSer^473^-Akt *SV40T-Large; PIG or SV40T-Large; PIG* -*INPP4B* MEF with quantitation plot of pSer^473^-Akt activation status (*n* = 3). *P*-values were determined using ANOVA. ^*^ *P* < 0.05, ^**^ *P* < 0.01, ^***^ *P* < 0.005.

To explore the relationship of *Inpp4b* deficiency on PI3K signaling in MEF, low-passage *Inpp4b*^*+/+*^ and *Inpp4b*^-/-^ *SV40T-Large* MEF were serum starved overnight and stimulated with epidermal growth factor (EGF). We consistently observed that *Inpp4b*^-/-^ MEF achieved higher peak levels of pSer^473^-Akt activation at 5–10 minutes compared to timepoints in *Inpp4b*^*+/+*^ MEF. *Inpp4b*^-/-^ MEF maintained prolonged peak levels of pSer^473^-Akt beyond 10 minutes of EGF and remained higher than *Inpp4b*^*+/+*^ MEF up to 30 min (Figure 6A). Conversely, *INPP4B* overexpression in *SV40 T-Large* MEF led to markedly decreased levels of pSer^473^-Akt after EGF stimulation. This decrease was observed at the peak pSer^473^-Akt concentrations of 5 and 10 minutes (Figure 6B). Together these results indicate that INPP4B regulates Akt activation dynamics in stimulated *SV40T-Large* MEF.

## DISCUSSION

Altered levels of *INPP4B* expression have been linked with cancer progression in various different human tumour types [42]. Previous works have demonstrated that *INPP4B* knockdown induced anchorage-independent cell growth, increased invasion and migration, and augmented Akt activation in immortalized HMECs [8, 9]. In contrast, other reports show that *INPP4B* overexpression enhanced proliferation and promotes anchorage-independent growth of HEMn-MP melanocytes and FHC normal colon epithelial cells [34, 38]. Herein, we present results which indicate that deficiency of *Inpp4b* in MEF can cooperate with very specific oncogenic alterations to induce cellular transformation. In our study, we observed that neither heterozygosity nor deficiency of *Inpp4b* were sufficient to alter growth characteristics of MEF on their own (Figure 1C). Spontaneous immortalization of MEF was also not observed in colony formation assays (Figure 1D, 1E). Similarity, *Inpp4b* deficiency was inconsequential for *H-Ras*^*V12*^ */E1A* induced cellular transformation of MEF (Figure 1F, 1G). *Inpp4b* deficiency did not inhibit *H-Ras*^*V12*^ */E1A*-induced cellular transformation as both *Inpp4b*^-/-^ and *Inpp4b*^*+/+*^ MEF displayed long-term growth potential consistent with immortalization and in an anchorage independent manner in the soft agar assay (Figure 1G, 1H). When co-expressed in MEF, *H-Ras*^*V12*^ and *E1A* potently induce cellular transformation due to the strong proliferative signaling through *H-Ras*^*V12*^ concomitant with inhibition of the *Rb* tumour suppressor pathway by *E1A* which bypasses Ras-induced senescence [44, 45]. *Inpp4b* deficiency did not cooperate with *H-Ras*^*V12*^ nor *E1A* oncogene overexpression to promote MEF transformation suggesting that *Inpp4b* may not play a role in *E1A* or Ras signaling pathways in MEF (Figures 2–3).

However, *Inpp4b* deficiency did promote *SV40 T-Large* mediated transformation of MEF, as demonstrated by an increase in anchorage independent growth and colony formation as compared to controls (Figure 4C–4D). Increased *SV40 T-Large* mediated cellular transformation was associated with elevated Akt activation as demonstrated by elevated pSer^473^-Akt after stimulation of starved MEF with EGF (Figure 6). Together, these findings are consistent with studies by Westbrook *et al.* [9] and Gewinner *et al*. [8] which demonstrated that shRNA knock-down of *INPP4B* in HMEC cells immortalized with *SV40 T-Large* and *hTERT* promoted cellular transformation. In addition, that *Inpp4a*^-/-^ MEF also displayed elevated pAkt levels compared to *wild typ*e after *SV40 T-Large* immortalization [46] support the notion that 4-phosphatase function is tumour suppressive in part through regulation of Akt signaling. Other mechanisms which have been linked to *INPP4B* loss include loss of ATM and ATR, both upstream regulators of the p53 pathway [22, 47] and INPP4B was reported to downregulate PTEN protein through its protein phosphatase activity [34]. Our efforts in this study demonstrated that p53 and Pten expression levels were unchanged between *Inpp4b*^*+/+*^ and *Inpp4b*^-/-^ SV40 T-Large MEF (Figure 4B) indicating that these mechanisms may not be associated with the phenotypes we observed in MEF.

Conversely, emerging evidence suggests that INPP4B overexpression may also promote tumourigenesis and cancer progression [32–36]. Thus, we tested whether *INPP4B* overexpression in MEF drives or cooperates with other transforming oncogenes in cellular transformation. We observed that *INPP4B* overexpression was unable to promote transformation in combination with *H-Ras*^*V12*^, *E1A nor SV40 T-Large* MEF. In fact, overexpression of *INPP4B* in *SV40 T-Large* MEF significantly decreased cellular transformation and concomitantly decreased pSer^473^-Akt levels (Figures 5, 6). These findings were reminiscent of those reported by Fedele *et al*. [10] in serum starved and EGF stimulated MCF7 at similar time points, where peak pAkt levels were lower after *INPP4B* knockdown, but at 30 minutes INPP4B overexpressing cells displayed slightly increased pAkt levels compared to the control [10]. Taken together, these findings highlight a tumour suppressive role for *Inpp4b* in the context of *SV40 T-Large* that coincides with elevated Akt activation and suggest that a functional alteration conferred by *SV40 T-Large* transduction cooperates with *Inpp4b* loss to promote cellular transformation *in vitro*.

In summary, our study aimed to address the role for *Inpp4b* in cellular transformation. Given the numerous contrasting models for *INPP4B* function in cancer, we investigated both deficiency and overexpression to elucidate predominant tumour suppressor or oncogenic roles, respectively in MEF. With respect to MEF transformation, we observed that *INPP4B* overexpression did not promote oncogenesis when combined *H-Ras*^*V12*^, *E1A* or *SV40-T-large* transduction. Although *Inpp4b* deficiency did not cooperate with overexpression *H-Ras*^*V12*^, nor *E1A*, we did observe that *Inpp4b* deficiency can increase the transformation potential of *SV40-T-large* transduction suggesting that *Inpp4b* may function in a tumour suppressive manner in this context, at least in part through its control of Akt activation. This suggests that an event specifically associated with suppressing *SV40-T-large* transformation is relieved by *Inpp4b* deficiency. Further investigation is required to elucidate the specific mechanisms at play.

## MATERIALS AND METHODS

### MEF preparation and culture conditions

Timed breedings were performed with C57BL/6J *Inpp4b*^+/-^ pairs and embryos were dissected from euthanized mothers at 13.5 days post-coitum. The skin fibroblasts were separated from the head and viscera and incubated in 2 mL of 0.25% trypsin for 30 minutes. The solution was then mixed vigorously by pipetting, followed by the addition of another 2 mL of trypsin and incubated at 37°C and 5% CO_2_. After 30 minutes, the trypsin was neutralized by adding 1 mL of fetal bovine serum (FBS), and re-suspended and grown in complete growth media (Dulbecco’s Modified Eagle Medium (DMEM) supplemented with 10% FBS and 1% penicillin/streptomycin) for 12–16 hours. Once the MEF had grown to confluency, the cells were frozen down and designated as Passage 1. Primary MEF were passaged every 3 days to not exceed 1.5 × 10^6^ cells per 10 cm dish. MEF were considered primary from Passage 1-Passage 4. All experiments were performed with primary MEF unless otherwise indicated.

### Genotyping

DNA was extracted by lysing the heads of the mouse embryos in DNA extraction buffer (1M Tris (pH8), 20% SDS, 0.5M EDTA, 5M NaCl made up in water) containing 10% proteinase K at 55°C shaking for approximately 16 hours. An equal volume of 100% ethanol was added and centrifuged at 21.9 × g for 10 minutes. The supernatant was discarded, and the DNA pellet was washed with 70% ethanol, dried completely, and re-suspended in 50 uL of nuclease-free water. PCR was performed using 2X FroggaMix (FroggaBio), to distinguish *Inpp4b*^*+/+*^ *, Inpp4b* ^+/-^ *, and* *Inpp4b*^-/-^. Genotyping primer pairs: wild type forward: 5′ GCTTCTGATAAAACATGGG 3′, wild type reverse: 5′ TGGGCACATTTATAAGCCTTC 3′, mutant forward: 5′ GCTTCTGATAAAACATGGG 3′, and mutant reverse: 5′ TGTTTTAAAAGCCTTGCTTGCTAAGTGTC 3′.

### Retroviral constructs

The following plasmids were purchased from AddGene: *pWZL hygro H-Ras*^*V12*^ (#18749), *pBabe-puro H-Ras*^*V12*^ (#1768), *pWZL hygro 12S E1A*(#18748), *and MSCV-puro-IRES-GFP* (#21654). *pWZL hygro SV40T-large* was a kind gift from Pier Paolo Pandolfi, and *12SLRC H-Ras*^*V12*^ */E1A* was a kind gift from Marisol Soengas. The *MSCV-PIG-*FLAG-*Inpp4b* was cloned by adding FLAG-*Inpp4b* to the *MSCV-puro-IRES-GFP* plasmid through Gibson Assembly (NEB).

### Retroviral transduction

For transfection of retroviral constructs, 3.0 × 10^6^ 293T cells were seeded on a 10 cm dish 24 hours prior to calcium phosphate transfection. Media was changed 3–4 hours prior to transfection. For each 10 cm dish, 10 ug of retroviral plasmid and 5 ug of retroviral packaging vector, pCL-Eco, were combined with 2M CaCl_2_ and made up to a final volume of 300 uL with sterile water. This DNA/CaCl_2_ mixture was vortexed while 300 uL of 2X HEPES-Buffered saline (HBS; 140 mM NaCl, 1.5 mM Na2HPO4) was slowly added to mixture. 600 ul of DNA/CaCl_2_/HBS mixture was gently pipetted onto 293T cells. Media was changed 24 hours after transfection and supernatants was collected at 48- and 72-hours post-infection. MEF were seeded at 8.0 × 10^5^ cells/10 cm dish 24 hours prior to infection. Fresh virus-rich media was collected and filtered using a 0.45-micron filter and added to respective plate along with 8 μg/ml protamine sulfate. Infections were repeated every 8 hours to increase infection efficiency. *PWZL-hygro* and *PWZL- H-Ras*^*V12*^ *-hygro* were used as negative controls, and coinfection with *PWZL-E1A-hygro* and *pBABE- H-Ras*^*V12*^ *-puro* were used as positive controls. *Wild Type* MEF cells were transfected with *MSCV-PIG-Inpp4b* and *PWZL- H-Ras*^*V12*^ *-hygro* to investigate INPP4B overexpression in cooperation with *H-Ras*^*V12*^ overexpression. *Inpp4b*^-/-^ MEF cells were transfected with *pBABE- H-Ras*^*V12*^ *-puro* to investigate the effects of *Inpp4b* deficiency in cooperation with *H-Ras*^*V12*^ overexpression on cellular transformation. Cells were selected for 4 days with hygromycin B at 75 μg/ml or 2 days of puromycin at 2 μg/ml selection, then cells recovered for 24 hours before plating for experiments. MEF infected with both puromycin and hygromycin B resistance plasmids were first selected for 4 days with hygromycin B at 75 ug/mL, allowed to recover for 24 hours and subsequently selected with 2 μg/ml puromycin; The cells were then allowed to recover for 24–48 hours before plating experiments.

### Growth assays

MEF were seeded at a density of 2.5 × 10^4^ cells per well in a 12-well plate for a 6-day growth curve. At each time point, cells were fixed with 10% formalin for 10 minutes and stored in phosphate-buffered saline (PBS) at 4°C. Once all time points were collected, cells were stained with 0.1% crystal violet, 20% methanol solution, then washed with water and dried for at least 4 hours. The crystal violet stain was solubilized by 10% acetic acid for 20 minutes and the absorbance was measured at 590 nm with Spectramax M3.

### Clonogenic assays

MEF were plated at 1000 cells per well in a 6-well plate and allowed to grow for 10–20 days without media change. Cells were then washed with PBS, fixed with 10% formalin and stained with 0.1% crystal violet, 20% methanol solution, washed with water and allowed to dry. Colonies were imaged and counted using Image J. Cell counts were normalized to the control group.

### Anchorage independence assays

200 mL of 6% Noble Agarose in sterile water was heated until liquified and brought to 42°C. Soft agar plates were prepared by mixing 2 mL of melted 6% Noble Agarose in MilliQ water in 18 ml of complete DMEM. 3 mL of 0.06% Noble Agar was plated per well of a 6 well dish and placed at 4°C to solidify. 1 hour prior to plating the dish was preheated in a 37°C cell incubator. 20,000 infected MEF were placed in 7.2 ul of media at 37°C then mixed with 0.8 ml of 42°C, 3% Noble Agarose in MilliQ water to obtain a final concentration of 5000 cells per well in triplicate of a 6 well dish in 0.3% Agar in DMEM. Colonies were stained using 0.05% crystal violet in 40% methanol. Colonies were counted using OpenCFU colony counting software (http://opencfu.sourceforge.net).

### qPCR

RNA was isolated using the Qiagen RNeasy Mini Kit, and subsequently treated with DNase. Reverse transcription was performed using the SuperScript IV VILO Master Mix (Invitrogen, 11766050). TaqMan Fast Advanced Master Mix (appliedbiosystems, 4444557). TaqMan probes for *Inpp4b* (Mm01247230_m1) and *actin* (Mm02619580_g1) were purchased from Thermo Fisher Scientific. All of the above conducted according to manufacturers’ instructions.

### Akt signaling assay

Passage 3–5 SV40 T-Large MEFs were seeded at 200 000 cells per well in 6 wells of a 6 well dish. After the MEF adhered to the bottom of the dish (4–6 hours after plating) the cells were washed twice with serum free DMEM then plated in starvation media (DMEM + 0.01% FBS). 16 hours later each well was allocated to the indicated time point (minutes). 100 ng/mL EGF was used to activate PI3K signaling. Activation was terminated at the indicated time points with ice cold PBS, followed by drying of wells, and subsequently flash frozen with liquid nitrogen and placed at –80°C prior to immunoblot.

### Western blots

Immunoblots were performed using standard conditions as previously published [32]. The following antibodies were used in this study. Anti-phospho-Ser^473^-Akt (NEB 14543); anti-pan Akt (NEB 29020); anti-β-Actin (NEB 4967); anti-FLAG (Abgent AP1013a-ev). Anti-INPP4B antibody detecting murine Inpp4b was a kind gift from Jean Vacher [30].

### Statistics

To compare means of 2 groups *p*-values were calculated using unpaired two-tailed Student’s *t*-test. For colony counts of the clonogenic assay and soft agar assay two-way parametric analysis of variance (ANOVA) was used to determine the significance value. A *P-value* < 0.05 was considered significant.

## Abbreviations

AML: Acute myeloid leukemia
E1A: Adenovirus early region 1A
EGF: Epidermal growth factor
H-Ras: Harvey rat sarcoma viral oncogene homolog
HMEC: Human mammary epithelial cells
INPP4B: Inositol Polyphosphate 4-Phosphatase, Type II
MEF: Mouse embryonic fibroblast
PIG: MSCV-Puro-IRES-GFP
OIS: Oncogene-induced senescence
PtdIns: Phosphatidylinositol
pAKT: Phosphorylated Akt
Ras: Rat Sarcoma
SGK-3: Serum/Glucocorticoid Regulated Kinase Family Member 3
SV40: Simian Vacuolating Virus 40
